# Perceptions of Technology and Digital Health Tools Among Recently Incarcerated Adults With Opioid Use Disorder: Semistructured Interview Study

**DOI:** 10.2196/90168

**Published:** 2026-09-17

**Authors:** Milan F Satcher, Elizabeth C Saunders, Kathleen D Bell, Sarah K Moore, Joshua D Lee, Lisa A Marsch

**Affiliations:** 1 Department of Community & Family Medicine, Dartmouth Health, 1 Medical Center Drive, Lebanon, NH, United States, 1 603-646-7014; 2 The Dartmouth Institute of Health Policy & Clinical Practice, Geisel School of Medicine, Dartmouth College, Lebanon, NH, United States; 3 Center for Technology & Behavioral Health, Geisel School of Medicine, Dartmouth College, Lebanon, NH, United States; 4 Department of Population Health, Grossman School of Medicine, New York University, New York, NY, United States

**Keywords:** user experience, criminal legal system, opioid use disorder, reentry, digital health, digital divide, mHealth, telemedicine, qualitative research

## Abstract

**Background:**

Individuals with opioid use disorder (OUD) who return to the community from incarceration face a high risk of fatal drug overdose. This mortality risk is related to significant barriers to accessing health care, social support, and resources needed to transition to and thrive within the community safely. Digital health tools have effectively supported substance use treatment and recovery, but their potential to support OUD during the high-risk period of reentry is underexplored.

**Objective:**

This study aimed to examine how adults with OUD returning from incarceration perceive and engage with digital health tools, and identify barriers and facilitators to their use.

**Methods:**

Semistructured interviews were conducted with a purposive sample of 39 adults recently released from New Hampshire prisons and jails. Participants were recruited from a randomized clinical trial (EXIT-CJS) comparing the effectiveness of extended-release buprenorphine, naltrexone, and enhanced treatment as usual on treatment retention. Interviews were audio recorded, transcribed, and analyzed using a deductive-inductive approach to content analysis. The COREQ (Consolidated Criteria for Reporting Qualitative Research) criteria were used to guide the study reporting.

**Results:**

Most participants preferred digital health over in-person care for both OUD treatment and other health care, mainly due to its convenience and resilience against reentry challenges. Yet, most participants also underscored the irreplaceable role of in-person therapeutic human connection in recovery and recognized this as a trade-off of stand-alone digital health. Participants described using digital health tools to overcome barriers that otherwise would have prevented them from receiving in-person care, such as transportation shortages, limited provider availability, and work scheduling conflicts. Accessing digital health was acknowledged as contingent on access to internet-capable devices, affordable connectivity, and digital literacy.

**Conclusions:**

The findings suggest that maximizing digital health’s impact in reentry will require a multipronged approach: investing in infrastructure to close the digital divide, addressing upstream structural barriers to accessing care, and partnering with impacted persons to co-design hybrid care models that enhance the feasibility of engaging in care during reentry while enhancing therapeutic human connection and respecting patient preferences.

## Introduction

Substance use disorders (SUDs) are highly prevalent in the US criminal legal system. An estimated 65% of incarcerated individuals meet the clinical criteria for an active SUD [1], and in 2019, approximately 15% of the 1.8 million incarcerated people had opioid use disorder (OUD) [2]. Upon release, individuals with OUD face an extremely high risk of fatal overdose—up to 40 times higher than that of the general population—driven by lost opioid tolerance, disrupted access to medications for OUD (MOUD), and poor coordination of medical and behavioral health care during reentry [3-6]. Accessing health care during reentry into the community is crucial, as individuals who experience incarceration also have higher rates of chronic diseases than the general population [7-9] and are less likely to receive treatment for these conditions during incarceration [10-12]. During this critical transition to the community, individuals must navigate a complex array of competing demands (eg, securing housing, employment, health insurance, and legal compliance) while facing stigma, surveillance, and legal exclusions that can further impede access to health services and supports [13-15]. In recent years, digital health technologies and services (eg, mobile apps, platforms, and telehealth) have rapidly advanced to support chronic disease management, including SUDs. These technologies are increasingly being used in addiction research and practice to deliver behavioral health interventions, monitor symptoms or substance use in real time, remind patients to take medications, and connect them with care providers or peer supports [16,17]. Digital health tools have a robust and growing body of evidence demonstrating their acceptability, feasibility, and efficacy and have shown promising potential to improve the assessment, treatment, and recovery supports for SUD [18-23]. Concurrently, internet-enabled devices (eg, smartphones, computers, and tablets) and the internet have become increasingly affordable and accessible to historically underserved populations, including those with SUD, in part due to federal programs that subsidize the cost of devices and data plans for persons with low incomes (eg, the Lifeline Telephone Assistance Program and the former Affordable Connectivity Program) [24-26].

Yet, for some groups, efforts to improve access to digital health technologies and the skills needed to use them have not kept pace with how quickly technologies are evolving [27,28]. Rural communities and populations subject to legally sanctioned discrimination face structural inequalities resulting from economic disinvestment, limited educational and employment opportunities, and inadequate health and social infrastructure [29]. Such systemic barriers constrain health and digital literacy, economic opportunity, and access to health-supporting resources. Incarcerated and reentering individuals with OUD are disproportionately impacted by such disadvantages [30-34], which may restrict their ability to access and benefit from digital health innovations despite their elevated health risks and needs.

Carceral facilities have a long-standing history of leveraging telemedicine as a cost-effective approach to overcoming their limited on-site medical services, including behavioral health services [35,36]. Beyond telemedicine, a small but growing body of research has examined the feasibility, acceptability, and/or effectiveness of various digital health tools for SUD among incarcerated populations, including computer-assisted self-directed therapeutic programs (eg, Therapeutic Education System), mobile apps, serious games, and U.S. Food and Drug Administration–approved digital therapeutics [37,38]. However, research on digital health tools specifically targeting reentry and especially among people with SUD remains nascent. Although nonspecific to SUD, a few reentry-specific studies have described the human-centered design of mobile apps, including personal health libraries [39] and cardiovascular disease support tools [40,41]. Reentry studies specific to SUD have identified the web-based Therapeutic Education System [42] and text-delivered peer recovery support [43] as feasible and acceptable during reentry. Another study designed and piloted a mobile health platform with linked interfaces for probation or parole officers and clients [44], demonstrating high usability and user satisfaction. There is a critical knowledge gap regarding the needed functions, design, delivery, and effectiveness of digital SUD interventions targeting the high-risk transition period of reentry.

Digital health tools have the potential to improve reentry health outcomes for OUD by supporting access to evidence-based treatments, improving communication between carceral and community providers, and delivering real-time support to patients during reentry. To design digital health interventions that are appropriate, acceptable, effective, and equitably accessible, it is essential to first understand how people with lived experience of incarceration, reentry, and OUD perceive these technologies and the determinants of access and engagement. This qualitative study aimed to assess how recently incarcerated adults with OUD perceive digital health tools and to identify barriers to and facilitators of engaging with these technologies during incarceration and reentry.

## Methods

### Study Setting and Population

This qualitative study was conducted at the New Hampshire (NH) site of the multistate randomized controlled trial, *Long-Acting Buprenorphine vs Naltrexone Opioid Treatments in CJS-Involved Adults* (EXIT-CJS) [45]. EXIT-CJS is an open-label, noninferiority trial comparing extended-release buprenorphine (XR-B, Sublocade), extended-release naltrexone (XR-NTX; Vivitrol), and enhanced treatment as usual among adults with criminal legal system involvement and moderate-to-severe OUD. Participants were either currently incarcerated with an expected release date within 6 months or living in the community with a history of incarceration or supervision within the prior 6 months. Participants in the medication arms were offered study medication injections monthly for 24 weeks post-release under the care of a local substance use treatment clinic, after which monthly study follow-up visits for survey assessments continued until week 48. This qualitative study recruited participants with a history of recent incarceration from the NH EXIT-CJS study sample. To be eligible for an interview, NH EXIT-CJS participants had to be at least 2 months post-release from jail or prison and still within the 12-month EXIT-CJS study period. Reincarcerated EXIT-CJS participants remained eligible once they had spent at least two cumulative months in the community and were still within the 12-month EXIT-CJS study window. The EXIT-CJS study launched during the COVID-19 pandemic, during which most partnering carceral sites increased their use of telehealth, including for the treatment of SUDs [46].

### Recruitment and Data Collection

To reduce in-person study activities during the COVID-19 pandemic, the NH EXIT-CJS study team gave all NH site participants a smartphone upon release with 1 year of unlimited talk, text, and data. Participants engaged in monthly EXIT-CJS study visits primarily via HIPAA (Health Insurance Portability and Accountability Act)–compliant Zoom to complete follow-up survey assessments. For recruitment into the interview substudy, the study team recruited incarcerated EXIT-CJS participants post-release during study visits 2 to 8 (weeks 4‐48) and recruited formerly incarcerated EXIT-CJS participants during the baseline study visit through the final week 48 study visit.

The qualitative study principal investigator (MFS) and the EXIT-CJS team met weekly to identify newly recruited EXIT-CJS participants who were eligible for an interview. The team aimed to recruit approximately 12 participants per EXIT-CJS study arm and purposively sample across the 3 study arms to maximize variation in biological sex, race, and ethnicity. During EXIT-CJS study visits with eligible participants, a team member described the qualitative study and provided an informational recruitment flyer. MFS then contacted interested persons via telephone to confirm eligibility, review study details, schedule Zoom teleconference interviews, and advise participating in a private setting. During the Zoom teleconference, the interviewer described the principal investigator’s professional background, shared motivations for conducting the study, and read aloud the study information sheet shared on screen. Interviewers assessed participants’ comprehension and addressed questions before obtaining verbal consent. Before beginning, interviewers assessed participants’ comfort with their level of privacy. Two female researchers conducted all interviews (MFS and KDB). Both researchers had completed training on qualitative interviewing. One researcher (KDB) engaged with some participants as part of the larger EXIT-CJS study team, while the other interviewer (MFS) had no prior relationship with participants. Interviews lasted approximately 90 minutes, were audio recorded, and transcribed verbatim by Rev.com. MFS reviewed the transcripts for quality and removed any potentially identifying information (eg, person names). Recruitment continued until thematic saturation was reached. Saturation was determined through team discussions assessing for emergence of new sentiments in interviews and reached by team consensus that no new themes were being expressed by participants. Participant recruitment for interviews occurred between August 2021 and February 2024.

### Interviews

The semistructured interview guide covered three domains: (1) substance use and treatment experiences post release, (2) medication and treatment preferences during reentry, and (3) perceptions of technology use for substance use treatment during incarceration and reentry (the focus of this analysis; Multimedia Appendix 1). Domains 1 and 2 were informed by the Dynamic Biopsychosocial Model and 2 prior qualitative studies on MOUD experiences during reentry, both of which were grounded in the Social Cognitive Theory[47,48]. The Dynamic Biopsychosocial Model considers how health is dynamically influenced by interactions between an individual’s biomedical, psychological, and socioenvironmental contexts [49]. Biopsychosocial factors and the weight of their impact on health change as individuals face new major life challenges (eg, reentry) that may demand different sets of resources. The 2 prior qualitative studies included (1) Velasquez et al’s study of reentry experiences and perceptions of XR-NTX among adults with OUD released from New York City jails [48], and (2) Fox et al’s study of reentry experiences and perceptions of sublingual formulations of buprenorphine among formerly incarcerated adults with OUD engaged in addiction treatment in New York City[47]. Velasquez and Fox drew upon the Social Cognitive Theory to ask questions about how these key determinants of behavior change (ie, self-efficacy, outcome expectations, perceived barriers and facilitators, behavioral patterns, observational learning, and the environment) influenced treatment engagement, retention, and post-incarceration opioid and other drug use [50].

Due to the lack of prior studies assessing technology use and perceptions among incarcerated and reentry populations, the team developed domain 3 interview questions drawing from the Dynamic Biopsychosocial Model, the domain’s research objective (ie, to assess how recently incarcerated adults with OUD perceive digital health tools and to identify barriers to and facilitators of engaging with these technologies during incarceration and reentry), and team member expertise in digital health research (ECS and LAM). Instead of pilot testing, all authors reviewed and revised the interview guide. Participants received a brief orientation to relevant technologies (ie, smartphones, laptops, and computers) and digital tools (eg, telemedicine and health apps) before discussing the third domain.

### Qualitative Data Analysis

Qualitative analysis of the third interview domain (ie, technology perceptions) used a deductive-inductive approach to content analysis [51] using Atlas.ti software. The coding team (MFS, ECS, SKM, and KDB) developed an initial codebook for this interview domain based on the Dynamic Biopsychosocial Model [49] and the concepts targeted in each interview question. The team coded small batches of 3 to 5 transcripts at a time, with analysts independently applying deductive codes and identifying new emergent codes. The team met regularly to revise codes and refine the codebook. Discrepancies in coding were resolved through discussion. The team applied a final code structure across all transcripts using a constant comparison method. Subtheme analysis involved grouping excerpts by code and developing categories. Codes were exported to separate Microsoft Word documents. For each code, 2 analysts independently reviewed the emergent categories across all code groups and synthesized these into overarching themes and subthemes, which were finalized through team consensus. The COREQ (Consolidated Criteria for Reporting Qualitative Research) criteria guided the reporting of the study methods and findings (Checklist 1) [52]. The team conducted the qualitative analysis from February 2022 to December 2025.

### Statistical Analysis

The NH EXIT-CJS team collected baseline survey data from all EXIT-CJS participants, including demographics, sentencing length, educational attainment, and health care engagement. The team calculated descriptive statistics (ie, counts with proportions and medians with IQRs) in Microsoft Excel to summarize these baseline characteristics for interview participants.

### Ethical Considerations

Ethical approval for the study was obtained from the Dartmouth College Institutional Review Board (STUDY00032371). The project was conducted in accordance with the Declaration of Helsinki guidelines and declarations. Verbal informed consent was collected from participants before each interview, and they were informed of their right to end the interview, pause, or skip questions at any time. The study team maintained participants’ privacy and confidentiality throughout recruitment, data collection, data analysis, and reporting. Participants remained deidentified using an assigned study number and removal of all mentioned names from interview transcripts. All data were stored securely on a HIPAA-compliant Google Drive maintained by Dartmouth College. Participants were compensated U.S. $50 via reloadable study debit cards for their contribution to the study.

## Results

### Sample Characteristics

A total of 40 EXIT-CJS trial participants were approached for an interview, among whom 39 agreed to participate, including 15 (38.5%) randomized to the XR-B arm, 11 (28.2%) randomized to the XR-NTX arm, and 13 (33.3%) in the enhanced treatment as usual arm. Table 1 describes the participant characteristics. Participants were predominantly men (n=23, 59%), non-Hispanic (n=37, 94.9%), and White (n=37, 94.9%). The median reported sentencing length was 328 (IQR 822.5) days. When asked an open-ended question in the interviews about access to internet-capable devices (Table 2), nearly half of participants (n=18, 46.2%) reported that the study-issued smartphone was their only functional internet-capable device, and only 11 (28.2%) participants expressed confidence that they would have been able to access a phone at release if the study had not provided one. Challenges to accessing a reliable device during reentry included uncertain access to items that were left in storage or placed under the care of others during incarceration, limited functional capability of their existing devices, the need to rely on public resources to access devices (eg, library computer and Lifeline-issued phone), limitations of borrowing devices from others, and device theft.

**Table 1.** Participant-reported demographic characteristics (N=39).

Characteristic	Values
Age (y), median (IQR)	36 (32‐39)
Sex, n (%)	
Female	16 (41)
Male	23 (59)
Ethnicity, n (%)	
Hispanic	2 (5.1)
Non-Hispanic	37 (94.9)
Race^a^, n (%)	
Black	1 (2.6)
Indigenous^b^	3 (7.7)
White	37 (94.9)
Other	1 (2.6)
Highest level of education completed^c^, n (%)	
Less than secondary school	2 (6)
Secondary school or GED^d^ completed	23 (67.6)
Tertiary, partial, and completed	8 (23.5)
Other (eg, technical or trade school)	1 (3)
Sentencing length (d), median (IQR)	328 (822.5)
Insurance coverage by type^e^, n (%)	
No insurance	5 (13.5)
Medicaid	31 (83.8)
Medicare	0 (0)
Private	0 (0)
Other	1 (2.7)
EXIT-CJS^f^ study randomization arm, n (%)	
XR-B^g^	15 (38.5)
XR-NTX^h^	11 (28.2)
ETAU^i^	13 (33.3)

a N=39. Total counts exceed 39 due to 3 participants reporting multiple races.

b Includes American Indian, Alaskan Native, Native Hawaiian, and Pacific Islander, combined due to small sample size and disclosure risk.

c n=34.

d GED: General Educational Development.

e n=37.

f EXIT-CJS: long-acting buprenorphine vs naltrexone opioid treatments in CJS-involved adults.

g XR-B: extended-release buprenorphine.

h XR-NTX: extended-release naltrexone.

i ETAU: enhanced treatment as usual.

**Table 2.** Self-reported uses of study and nonstudy issued internet-capable devices.

Use type	Examples
Communication	Texting, calling, video chatting, and email
Information seeking	Searching for health-related information (eg, medication side effects), local services, GPS navigation
Daily task management	Calendar, reminders, online shopping, managing appointments
General health support	Telehealth, patient portal, health trackers (ie, fitness, sleep, mood, and health behaviors), and meditation apps
Recovery support	Online groups (ie, mutual help, Alcoholics Anonymous, Narcotics Anonymous, and SMART^a^ Recovery), substance use trackers (eg, Clean Time app), telehealth appointments with MOUD prescribers, and behavioral health counselors
Entertainment and social connection	Streaming TV, videos, music, social media, games, gambling, dating apps, and communication with family and friends
Finance management	Apple Pay, Venmo, PayPal, and banking apps
Activities to address social needs	Job applications, rideshare, education (eg, online courses and tutorials), and income generation (eg, eBay sales and survey completion)

a SMART: Self-Management and Recovery Training.

### Theme 1: Preferences for In-Person Versus Digital Care Modalities During Reentry to the Community

#### Overview

Participants expressed a range of preferences for in-person versus digital care modalities during reentry, with some expressing neutral attitudes, but most preferring hybrid models of care to leverage digital conveniences while maintaining some level of in-person connection. Participants described types of health care they preferred to receive via in-person or digital care modalities.

#### Neutral

Several participants lacked a strong preference for care modality during reentry. Among these participants, some perceived no important differences between in-person and digital health care, stating:

*that’s about as effective as going in person except for having to drive there and parking and all that crap.*

[Participant 020, 39M]

One participant also perceived the quality of care as equivalent, explaining:

*I think you would get the same quality of sit down with a person regardless.*

[Participant 031, 31M]

Another participant asserted that the experience would be different, without impacting quality:

*It would be different. They’re [the providers] not physically there, so yeah, it’s different. I don’t think it would be any better or worse per se, but it is definitely different just in general nature of it*.

[Participant 024, 32M]

One participant’s neutral stance was driven by willingness to engage in any modality that benefited his health and recovery:

*For me, I feel comfortable, if it’s for my own good, it don’t matter how I have to do it*.

[Participant 027, 49M]

Similarly, another participant emphasized that accessing care was the priority, expressing willingness to use any available modality, stating:

*I feel like going in person to counseling is nice, but again, [digital healthcare is] convenient... If I had to do counseling over Zoom or whatever, it’s better to do it than not be able to make it to an appointment or whatever. I could break my foot and have to be laid up in bed and I’d still have access to treatment, or counseling, or psych, my PCP.*

[Participant 023, 33F]

#### Hybrid Models Preferred

Most participants expressed appreciation for both in-person and digital health care and a desire to integrate them, with one participant stating:

*It would be a mix, I think, a mix of if you had some in person and then some online, would be ideal*

[Participant 032, 39M]

One participant described distinct use cases for in-person versus digital health care, indicating a desire to engage in both modalities in different ways:

*I feel that for some things it’s important to be face-to-face with your doctor. But things like this [telehealth visits], I like it because not everybody has transportation to get to certain places that are far away. So being able to meet online and on Zoom and stuff like that is very helpful... Counseling or therapy or whatever. Maybe just an update or a quick talk with the doctor or whatever. As long as you don’t have any test results or exams done or anything, I’d be fine with it.*

[Participant 036, 38F]

Another expressed a desire to leverage the advantages of both modalities, stating:

*[digital healthcare] it lacks the connection piece a little bit. Not extremely. You can definitely still have that connection. I think the best way to have that [connection] is to have in-person meetings, and then on the phone as well... Because as far as—especially for me—without transportation to doctor’s appointments and stuff like that, it’s definitely huge for me to be able to just do it over the phone.*

[Participant 013, 23M]

#### In-Person Preferred

Participants predominantly preferred to engage in health care via in-person encounters for group-based behavioral health services, intensive outpatient programs for SUD, acute medical treatment, and encounters with a new health care provider. Only 2 of the 39 participants had a strong personal preference against engaging in digital health care, which they perceived as impersonal, less effective, too convenient, and easy to manipulate compared to in-person care. As one such participant stated:

*It’s super impersonal... you can’t read somebody through chatting with them on your iPhone. It’s easier to catch somebody lying or something like that if you’re in person with them... I just prefer to actually speak to somebody in person.*

[Participant 039, 25F]

The other participant who strongly opposed digital health care emphasized that convenience is not always a benefit and described the value of embodying actions in person in support of his recovery, stating:

*For me, it’s just too impersonal and too convenient, too. I could just sit or be anywhere I’m at and turn the thing on and not have to do anything proactive in my thing. There’s something to the effect of making an appointment, getting in a vehicle, driving to it. It’s just there’s so much more action in it. And this is just very inactive... It didn’t take me anything to go and do what I had to do right now. And so, it really doesn’t hold too much of a value to me. If I care enough about something, then I can put in some effort and get to where it shows something... It might even work to the spirit of things.*

[Participant 001, 43M]

Other participants who preferred engaging in health care in person emphasized the value of human connection as facilitating their sense of knowing their health care providers and/or fellow program participants and being known by them. As one participant shared:

*I go to my primary care doctor in person because like I said, he’s more than a doctor to me because he sits there, talks to me about my whole life... he is really good to go talk to him because he has good insight... I’ve been with him 10 years and I've only been sober three or four of it, so he knows how I am.*

[Participant 014, 42M]

Many regarded the accountability received during in-person encounters as critical during early recovery. As one participant expressed:

*I think in-person meetings like IOP [intensive outpatient program] and stuff makes you get up and go and keeps you present in the group. If you just do it over the phone, you don’t have to be present. You can be sitting in somebody’s car with a car full of people being quiet with the phone muted. You can be high. And nobody’s going to know. I think IOP is meant to be in person... because part of the IOP is being present, part of AA [alcoholics anonymous] meetings is being present, claiming your seat.*

[Participant 028, 43F]

Many participants also described how in-person care enables positive reinforcement of healthy behavior changes and facilitates the building of social capital for recovery. As expressed by one participant:

*I saw people doing the AA meetings and NA [narcotics anonymous] meetings on Zoom. It’s good, but... a big part of doing those meetings is like, ‘All right. The first chip is 1 to 28 days or a month. You got two months clean.’ A lot of people get up, and they clap. You get up, you go get your coin medallion key tag, whatever it is. You shake the person that presented it to you, give them a hug, fist bump, whatever. You’re not doing that over the phone. It’s a totally different vibe, atmosphere.*

[Participant 026, 38M]

Some participants also perceived in-person encounters as more effective than digital health care, in part because of more accurate in-person assessments of substance use and mental health status during early recovery:

*I do see... [therapist name] right now for therapy... sometimes she’s like, “Oh, you seem pretty relaxed and calm and not fidgety.” And it’s like, I really will be fidgety. It’s just usually with my legs and my feet. So, it’s not something that can be seen really.*

[Participant 016, 36F]

and

*I think sometimes a con for [digital healthcare] could be people trying to hide what’s really going on, or whereas if you were in person, sometimes when you know someone, you know that they’re not right... Say I am in a depression, and you see me from the waist up and I look put together, but maybe in real life I’m [not].*

[Participant 033, 48F]

#### Digital Preferred

Among participants who preferred digital modalities for health care delivery, they were willing to receive individualized counseling for mental health or SUD, medication management appointments, and group-based SUD treatment via digital delivery. Convenience was the most cited reason for preferring digital health care. Because participants faced numerous logistical challenges and competing demands during reentry, participants appreciated the convenience of digital health care and detailed secondary benefits, including not needing to miss work to attend appointments, saving money on transportation or lost wages, time saved by avoiding travel, on-demand access to care, more efficient appointments, enhanced quiet and privacy by engaging care from a self-selected safe space, and reduced exposure to substance use risk factors (eg, witnessing active use or drug sales near treatment sites). As one participant described:

*I would’ve preferred it [behavioral health appointments via telehealth]. Just the inconvenience of having to make an appointment. It’s like, taking time off from work, and then getting there... I try and do it first thing in the morning or late-late in the afternoon, and it never works out that well. So, it’s like, by the time I get out of there, the whole morning’s shot, or if it’s an afternoon one, I can work the morning, and then knock off at like 1:00 to make it for a 2:00 or 2:30 appointment, which would be very difficult for me right now, because I’m working an hour and a half, two hours one way. So, if I have an appointment, it really messes up the schedule... I don’t think it [digital healthcare] would be less effective. I only see it as a good thing. I see nothing negative about it.*

[Participant 006, 50M]

Participants also highlighted how digital modalities helped them access care despite facing resource limitations that otherwise prevent access to in-person care settings. As one participant expressed:

*Where I don’t have the license, especially, it’s made it a lot more convenient and I love it. It’s helped a lot, having it on my phone.*

[Participant 011, 34F]

Several participants perceived digital health care as a resource to expand their health care options and increase their ability to exercise treatment preferences in a way that is not possible locally through in-person care. As one participant stated:

*I think it would spread a way bigger options of providers... I’m not limited to just these two frigging doctors that had openings in [NH city name]. Or if I wanted to find somebody that I got along with better from California or something, I could do that recovery-based [care], so I don’t have to go to their office.*

[Participant 012, 39M]

### Theme 2: Barriers to Engaging in Digital Health

#### Technology Access and Infrastructure

Regardless of stated preference for or against digital health care, several participants highlighted the challenge of accessing the internet and internet-capable devices during reentry, which may limit access to digital health care (Multimedia Appendix 2). Several participants described options for accessing free internet-capable devices, including old phones that they owned prior to incarceration, devices loaned by the public library, free phones from community organizations, or government-sponsored phones (eg, Lifeline and SafeLink) but emphasized the low quality, limited functionality, and unreliable performance of such devices. As one participant shared:

*I was given a government[-sponsored] phone... but it only had a limited amount of data, limited amount of call time. It was no memory on it. Anything I’d try to save, I’d have to save basically everything to my e-mail or to my Facebook, so that way I could have access to it later. I’d just send myself stuff on Facebook Messenger, but any other app that I wanted to download, I couldn’t have. I couldn’t have put Zoom on there. That would not have worked, or Google Meet. That would not have worked because it did not have enough data, or enough memory space, or whatever. The service was, if it was no Wi-fi, then it was pretty much useless.*

[Participant 032, 39M]

Participants also cited data costs as a barrier to accessing the internet consistently, stating:

*The free [EXIT-CJS study] phone [and 1-year data plan] definitely helps because if you’re just coming out and transitioning, it’s kind of overwhelming to remember to pay your cell phone bill or even have financial burdens more than what you already have accumulated before you went in.*

[Participant 023, 33F]

#### Digital Literacy and Comfort

Participants expressed discomfort with using technology related to its technical complexity, stating:

*There’s also so many extra steps to it too... Taking pictures and sending things and getting back. And I just get aggravated with it.*

[Participant 001, 43M]

Even some participants who preferred digital health care described experiencing anxiety related to unfamiliarity with technology, particularly during initial digital health care encounters. As one participant shared:

*Just at first it [telehealth] was nerve wracking. You don’t know if the person’s going to treat you properly just because they can’t see you [in person], but they can see you [online].*

[Participant 032, 39M]

Participants also described the lack of exposure to technological advancements during incarceration as a driver of discomfort and limited digital literacy. Another participant highlighted how prolonged incarceration causes individuals to fall behind on digital literacy, stating:

*I went away to prison... in 2008. I didn’t get out until 2014. The big thing when I went away was the Razer phone, the slide phones. There was no smartphones. There was no Facebook. When I got out, I didn’t know how to dial a telephone. You’ve got to remember that some of these offenders are in for 10 years or more... I might be savvy with technology, but there’s a lot of people that aren’t, and there’s older people that are released too.*

[Participant 009, 37F]

#### Privacy and Security Concerns

A minority of participants expressed strong concerns about privacy, confidentiality, and security. As one participant expressed concerns about unknown or unauthorized surveillance:

*It’s not even like it’s private. I don’t care what anyone says, how many things that are... I don’t believe that our stuff’s not monitored.*

[Participant 001, 43M]

Others described the risk of data breaches and information theft (Multimedia Appendix 2). For such participants, physical health care offices were perceived as providing superior privacy:

*I could just go to my counselor’s office and have the privacy of being in the office with my counselor.*

[Participant 017, 23M]

### Theme 3: Drivers to Engaging in Digital Health

#### Access to Quality Devices and Internet

Many participants described quality internet-capable devices as an essential resource to reliably access digital health care. One participant explained:

*If they’re [Internet-capable devices] provided, like you guys provided me with a cell phone... it could make things easier. Because a lot of these guys don’t have transportation, so it’s kind of like a catch-22 sort of situation... I think that it [healthcare] could be done over the phone, and providing someone with a cell phone is easier than providing them the transportation every week, if you ask me.*

[Participant 024, 32M]

Participants also highlighted free data coverage as a resource that alleviates financial barriers to engaging in digital health care.

#### Ease of Use

Digital health care was generally regarded as easy to engage in, even among participants who preferred in-person care. Several participants contrasted the ease of engaging in digital health care to the logistical complexity of accessing in-person care, especially while facing numerous challenges and barriers during reentry. The logistical ease of digital health care also reduced cognitive demands for some participants and supported on-demand access to care, as expressed:

*“I know that my insurance provides transportation, but sometimes you don’t think about it three days ahead of time. It just is a last-minute thing and then you’re screwed.*

[Participant 033, 48F]

#### Integrates Into High Competing Demands, Low Resource Context of Reentry Without Added Burden

Participants emphasized the resource constraints experienced during reentry, including housing challenges, transportation limitations, debt and financial strain, lack of paid time off for medical appointments, and strained social support systems. The convenience of digital health tools was described nearly universally, even among the 2 participants who strongly preferred to engage in health care in person. Digital health tools were described as making it possible to access care at times when competing demands during reentry would have otherwise made health care inaccessible, such as during times without transportation, conflicting work schedules, or lack of child care. One participant described:

*I feel like maybe at that time [reentry] would be more convenient to do it over a phone or computer, just because when I was coming in, reentering, I didn’t have a car and a certain schedule from the halfway house, so it would’ve been a lot easier to do stuff over that than in person at that time*

[Participant 019, 35M]

Participants perceived digital health care as a means to avoid incurring the resource costs associated with accessing in-person care (eg, lost wages, transportation costs, time, and strain on social support’s resources). Digital health care was also described as integrating more seamlessly into the precarious support systems they had carefully assembled, enabling access to care while reducing the risk of disrupting the balancing act of tangible and relational resources they had negotiated access to during reentry. As exemplified by one participant:

*Most people when they get out, they ain’t got a license, they’ve barely got a place to live. So, this way it would be a lot easier and probably give them less stress too... Before this I had to go to [Town A], [Town B], [City C for healthcare], you had to go everywhere and it gets overwhelming in between work and stuff because then you’re missing all that time from work too... My boss drives [me] to [out of state site]. It’s not like I can just jump in a car and drive to [out of state site] to meet him. So, if I have my phone with me, I can jump on the phone and just do it [digital healthcare].*

[Participant 014, 42M]

Participants who had experienced past stigma from health care providers or hypervigilance and anxiety during reentry appreciated their greater control over time and place of engaging in digital health care, stating:

*“I think that it’s [digital healthcare] sometimes better because I get serious anxiety going to the doctor... I get really worried for some reason, just being there. You’re just sitting in a room and they go in and out and they shut the door, and you don’t know who’s going to come in and you don’t know what they’re going to say. You don’t know if they’re going to take you and just be like, ‘You’re going to get locked away somewhere,’ or something. I don’t know. It’s not a rational fear, but it just plays in your head. In my head, anyway. Having to set the appointments and then just talking to people in person, having to be there. You’ve got to be worried about how you’re dressed. You’ve got to get, getting there.*

[Participant 032, 39M]

Yet many participants also acknowledged the loss of synchronous, in-person human connection as an undesired trade-off of convenient digital health care, stating:

*[digital healthcare] lacks the physical connection... I definitely find it more comfortable being in a room, even though I’m really young and I love technology, I find it very resourceful and everything, but definitely I’ve had both experiences, on the telephone or on Zoom and stuff like that, compared to being in the room. And I just think over the years I’ve definitely found a lot more connection with the actual provider in the room. But also, it’s a lot easier to do it on your phone. So, you don’t have to make CTS rides [Coordinated Transportation Solutions for individuals on Medicaid and/or Medicare], you don’t have to go. So, there’s definitely pros and cons to it.*

[Participant 013, 23M]

## Discussion

### Principal Findings

In this qualitative study of recently released adults with OUD, participants described digital health tools as an acceptable modality for engaging in health care during reentry. Only 2 of the 39 participants expressed strong personal preferences against engaging in digital health care. However, even these participants viewed digital health technologies as highly convenient and easy to integrate into their lives amidst the abundant socioecological challenges encountered during reentry. Most participants emphasized that the digital delivery of health care reduced or eliminated many of the practical barriers that routinely block access to in-person care during reentry, including transportation costs and logistics, lost wages for time off work, and fragile social supports. In doing so, digital health technologies allow health care to be delivered any place and any time that participants could feasibly receive it and enable more autonomy to act on their care preferences. Digital health technologies support care models that meet reentering patients where they are rather than requiring them to overcome excessive resource constraints to reach care.

### Interpretation

Our findings align with prior evidence that telemedicine and other virtual care modalities can increase care access and patient satisfaction and overcome travel-related barriers, particularly for populations facing geographic or resource constraints [53-55]. The high acceptability of digital health during reentry suggests the potential to develop digital health tools that target reentry-specific overdose risks and health needs, such as delivering reentry planning and resource mapping, overdose prevention interventions and education, and linkage facilitation to addiction treatment and recovery supports.

However, participants emphasized that the benefits of digital health care are contingent on a set of enabling resources: a reliable internet-capable device, an active data service, the financial means to sustain connectivity, and a baseline of digital literacy and comfort with technology. These findings are consistent with studies assessing the feasibility of engaging in digital health for OUD [55]. Without these resources, digital care is selectively accessible and risks exacerbating disparities among people reentering the community [28]. The risk of widening the digital divide is particularly relevant for settings with limited broadband infrastructure, such as rural communities in the NH study setting, as well as during periods of public disinvestment in connectivity supports. For example, the recent wind-down of the Affordable Connectivity Program eliminated a widely adopted subsidy that reduced the cost of internet-capable devices and broadband service for low-income households [24,56]. Our study adds to the well-documented literature on the digital divide among marginalized populations by highlighting the unique vulnerabilities and contexts that shape access to digital health care specific to adults with OUD during reentry and the need for investments in connectivity, device access, affordability, and knowledge transfer to realize equitable benefits.

Participants nearly universally described a high value for in-person, human connection when receiving health care, even those who preferred digital care for convenience. Consistent with findings in the general population, in-person care was seen as important for facilitating diagnostic accuracy [57], and prosocial human connection was regarded as therapeutic and positively reinforcing. Some participants described the tension between desiring in-person care and the perceived higher level of accountability but lacking the resources necessary to participate in an in-person care delivery model. This emphasis on human connections is consistent with literature linking social capital and healthy interpersonal support to recovery outcomes [58-61], and it suggests that any digital health care models that wholly eliminate in-person connection and human presence may risk eroding important recovery supports. This signals a need to address the upstream structural barriers to reaching in-person care models during reentry, such as restricted access to adequate housing and employment, limited transportation, debt, and insufficient financial assistance [30,31,62], which may go unaddressed and therefore be reinforced through a focus on digital innovation alone [63,64]. Structural reforms in combination with care model redesigns that make care less provider-centric and more patient-centered may strengthen patients’ capacity to feasibly engage in care in person.

Participants’ accounts also indicate the desirability and potential acceptability of hybrid models that intentionally blend digital tools with preserved or enhanced human touch. These models rethink where, how, and when we deliver in-person services to align with patients’ existing resources for access instead of the current model of providing access contingent on patient resource acquisition. One example of a potentially acceptable model being tested with patients with OUD, including reentry populations, is the InMOTION mobile pharmacy and clinic, which brings a low-barrier mobile SUD and infectious disease treatment into community settings via hybrid in-person and telehealth encounters [65]. Such a model may provide a practical template for patient-centered hybrid care approaches. Other potentially acceptable models not yet tested with incarcerated and reentering populations may include device distribution programs or telehealth access points located in accessible, trusted, community-based locations (eg, community centers and pharmacies) equipped with the necessary technologies, which might extend access where connectivity is unreliable, as has been demonstrated in other populations [66,67]. Future research should explore design features of digital tools that preserve and strengthen therapeutic human relationships.

A less commonly expressed but notable sentiment expressed by a few participants included the role of internalized and enacted stigmas in shaping preferences for in-person versus digital health care. Regarding internalized stigma, 2 participants described recovery as necessarily effortful, made valid by the exertion of willpower, and characterized by experiencing triumph amid adversity. These sentiments reflect the broader discourse on willpower-driven recovery and biases against approaches perceived as requiring less willpower (eg, MOUD), which can be dismissed as a “crutch” not representative of true recovery [68,69]. The internalization of the willpower-over-adversity recovery trope may discourage some people with SUD from engaging in emerging resources that reduce the effortful burden of recovery (eg, digital modalities of care delivery), regardless of their effectiveness. Regarding enacted stigma, 2 participants who preferred digital health care described how it has helped to avoid experiencing stigma from health care staff when circumstances make it impossible to adhere to traditional care norms and expectations (eg, arriving on time when Medicaid ride is unpredictably late). Previous studies have described how enacted stigma by health care providers can negatively impact care experience and engagement for people with SUD [70-72]. Altogether, our findings suggest that digital interventions for SUD may benefit from integrating stigma prevention and mitigation strategies to facilitate reach, adoption, and engagement.

Finally, policy levers may provide a pathway to address the digital divide for reentering populations on a wider scale. Payers and Medicaid programs should examine the cost-effectiveness of covering reliable internet-capable devices and data plans (eg, for the first year of release from incarceration). The Centers for Medicare & Medicaid Services and states should also consider using Medicaid 1115 demonstration applications to pilot coverage of internet-capable devices and data plans during reentry and explore innovations to address connectivity supports, other social determinants of health, and digital care models. Other potential state-based funding mechanisms to support digital access and innovation during reentry include Rural Health Transformation Funds and Opioid Abatement funds. Such policy levers could directly address the resource barriers our participants identified and help ensure that digital solutions reduce rather than exacerbate reentry disparities.

### Limitations

The findings are based on a purposive sample of adults with OUD released from NH prisons and jails, recruited from participants in a randomized clinical trial. Leveraging trial recruitment maximized the information obtained from existing participants, enhancing the return on taxpayers’ investment in research. However, several limitations exist. As a qualitative study, the findings reflect reentry experiences within the NH context with limited relevance for dissimilar populations or contexts. All participants were recruited from NH, where the population is predominantly White. While Black adults are disproportionately incarcerated in the state [73], our team was unable to recruit participants from racially and ethnically minoritized backgrounds. The lack of racial and ethnic diversity in our sample misses relevant perspectives, particularly given the overrepresentation of Black and Brown individuals in carceral systems nationwide due to systemic racism in policing, sentencing, and related structural inequities [74]. Our sample also had prior exposure to digital devices, including access to tablets during incarceration at the NH Department of Corrections, receipt of smartphones from EXIT-CJS, and experience participating in virtual study visits with the EXIT-CJS study team. Many of EXIT-CJS’s partnering carceral sites increased the use of telehealth during the COVID-19 pandemic, providing additional potential exposure to digital health care [46]. Therefore, this sample is not representative of reentering populations who have limited experience engaging with technology. Our study also describes perspectives provided over a 2.5-year period due to slow recruitment related to the larger trial’s recruitment pace, the substudy’s narrower eligibility criteria (ie, recently incarcerated only, not inclusive of community supervision alone), and attempts to balance recruitment across all 3 study arms and recruit both sexes. Given the fast pace of technological advancement and improving accessibility, later recruits may have experienced different levels of exposure to technology than earlier participants.

### Future Research

Future digital health research with incarcerated and reentering populations should intentionally include more diverse geographic and demographic samples to enhance the information power of the findings to reflect the needs and priorities of impacted communities. Such research should also intentionally recruit from more technology-naïve populations who have limited-to-no access to technology during incarceration or reentry to characterize their perceptions, preferences, and needs. Finally, our study launched before the mainstream emergence of AI and therefore did not assess perceptions of this new technology. In addition to enhancing personalized intervention and education on overdose prevention and substance use treatment, AI may present novel opportunities to address digital literacy needs among incarcerated and reentering populations. For example, closed-network AI models in correctional settings could provide interactive, self-paced training on basic and emerging digital tools without requiring open internet access. Such approaches could help prepare individuals for the technological advancements they will encounter post-release, reducing one barrier to engagement with digital health. Future research should assess this population’s perceptions of AI tools and ideas for innovation.

### Conclusions

Adults with OUD returning from incarceration regard digital health as a valuable tool for expanding access to care and overcoming structural barriers to care during reentry, while emphasizing that its benefits are both contingent upon reliable, affordable access to devices and data and at odds with the strong desire for in-person therapeutic human connection. To realize the promise of digital health for improving health care access and usage during the high-risk reentry period at scale, stakeholders must partner with impacted communities to co-design acceptable and accessible technological innovations [75-78] in combination with meaningful investments in connectivity, device access, digital literacy, and hybrid care models that preserve interpersonal connection. Without these complementary investments, digital modalities risk reinforcing existing inequities among incarcerated and reentering populations rather than ameliorating them [79].

## Supplementary material

10.2196/90168 Multimedia Appendix 1 Interview guide.

10.2196/90168 Multimedia Appendix 2 Subthemes and illustrative quotations exploring barriers and drivers to engaging in digital health during reentry.

10.2196/90168 Checklist 1 COREQ checklist.

## Abbreviations

COREQ: Consolidated Criteria for Reporting Qualitative Research
EXIT-CJS: Long-acting buprenorphine vs. naltrexone opioid treatments in CJS-involved adults study
MOUD: medications for opioid use disorder
OUD: opioid use disorder
SUD: substance use disorder
XR-B: extended-release buprenorphine
XR-NTX: extended-release naltrexone
