# Supplementary material for: Perceptions of Technology and Digital Health Tools Among Recently Incarcerated Adults With Opioid Use Disorder: Semistructured Interview Study
Source: J Med Internet Res. 2026 Sep 17;28:e90168. doi: 10.2196/90168 (PMC13585019; doi:10.2196/90168)
Supplement: Supplementary file 2 [file jmir-v28-e90168-s001.docx]

**Potential for Using Digital Health Tools in Substance Use Treatment**

Thank you for sharing with me about your life, substance use, and medical care experiences since leaving prison/jail. In this next set of questions, I will ask you to imagine what it would be like to receive health care through devices like a smartphone, tablet, or computer. I'm just going to share my screen.

*(Share screen to show examples of telemedicine and mobile apps on PowerPoint)*

You may be familiar with all these technologies, but I review them with everyone just in case. This is a picture of a smartphone, computer, laptop, and tablet. *(Arrow over each image as you name them)*

People can use these devices to receive different types of healthcare. One example is telemedicine. Telemedicine means a medical visit with a provider, such as a doctor or a counselor, using video over the Internet, similar to what we're doing right now. Another way of receiving healthcare through devices are through apps. This is an example of an app, called MyChart. It allows patients to connect directly to their medical record. They can see their past and upcoming visits. They can send messages to their providers. They can see their test results and request medication refills.

Another example is called Quit Start. It's a free app for people who want to quit smoking and provides support in their smoking cessation efforts. For example, people can indicate how they're feeling, such as if they're having a craving or if they slipped and smoked, if they're feeling low in their mood. Depending on what they select, the app provides tips that are customized to what they're currently experiencing. Patients can also track their progress. They can set a quit date and see how long they they've maintained it. They can track how much money they've saved by quitting. They can track cravings and slips.

These are just a few examples.

You were given a smartphone by the EXIT-CJS research study. Besides that smartphone, do you use anything else to connect to the Internet (e.g. computer, tablet)?

What kinds of activities or tasks do you do on the *device mentioned*?

Probes:

Do you use any apps like task management apps, calendar, reminders, Maps, email?

If the EXIT-CJS study hadn’t given you a cell phone, would you have had access to a phone when you were released?

What do you think about receiving healthcare on a smartphone or computer (i.e., seeing your doctor or getting health advice)?

Probes:

Have you ever received healthcare in this way?

**If yes:** How was that experience for you?

What did you like vs. not like about it?

Any concerns that you had?

**If no:** If given the option, would you receive healthcare in this way?

What would you like vs. not like about it?

What concerns would you have?

What kinds of health care would you feel comfortable receiving in this way? (e.g., general medical care, substance use treatment, pharmacy, counseling, etc.)?

What kinds of health care would you prefer not to receive in this way?

Do you think receiving substance use treatment on a computer or smartphone while you’re returning to the community would be any different from receiving in-person substance use treatment? If so, in what way? (i.e., more or less effective, convenient, private, isolating)

Probes:

How would receiving substance use treatment on a	computer or smartphone impact your overall reentry experience?
