# Supplementary material for: Perceptions of Technology and Digital Health Tools Among Recently Incarcerated Adults With Opioid Use Disorder: Semistructured Interview Study
Source: J Med Internet Res. 2026 Sep 17;28:e90168. doi: 10.2196/90168 (PMC13585019; doi:10.2196/90168)
Supplement: Supplementary file 3 [file jmir-v28-e90168-s002.docx]

**Multimedia Appendix 2.** Subthemes and illustrative quotations exploring barriers and drivers to engaging in digital health during reentry

| **Theme and Subthemes** | **Illustrative Quotes** |
| --- | --- |
| **Care Modality Preferences** | |
| In-Person Preferred | *“[Digital is] a lot more convenient, but at the same time, the amount of time people spend on their phones nowadays is disgusting. So even though it's more inconvenient to physically go to an appointment, I prefer to do that rather than take the super easy route where I can just sit on my ass and get what I need.” (Participant 039, 25F)* |
| Digital Preferred | *“I'm all for that [digital healthcare] because that cuts out having to get transportation and being on time… so leaving jail, if you were to have all these things [digital SUD treatment, appointments] in place and not have to worry about getting a phone… that's huge… I could only see positive things coming from that, but there are also negatives if you're not going to do the right thing with that device or limited resources for that.” (Participant 030, 37F)* |
| Hybrid Preferred | *“Early on in treatment, it probably would've been more beneficial to see somebody face-to-face, because they can see your physical appearance a little bit better, rather than over a Zoom…* *I probably wouldn't have ODed if that [digital SUD treatment during reentry] was the case, because I would've been able to get into somewhere very quickly if it's in the palm of your hands.” (Participant 038, 42F)* |
| Neutral | *“No, you get the same experience…” (Participant 035, 33M*)  *“I'm kind of neutral about it.” (Participant 036, 38F)* |
| **Barriers to engaging in digital health** | |
| Technology access and infrastructure | *“[Digital healthcare] would be more convenient, I guess, if they had that capability. They have to be given the capability to be able to do that. A lot of people getting out of jail and prison don't have cell phones or don't have a tablet or whatever. So that could make things more difficult.” (Participant 024, 32M)*  *“[Study phone] It’s just reliable for one… it adds so much more than, say, those...* *the Assurance Wireless or those government phones like the Obama Phone. It's just some of those phones aren't equipped to... or the speed of them as far as trying to get in contact with someone, the email, or... I don't know. This [study] phone is definitely a nicer phone, for sure.” (Participant 026, 38M)*  *“I would have had a phone, but it wouldn't have data on it. I would have to connect to Wi-Fi where there was Wi-Fi, if there was Wi-Fi.” (Participant 032, 39M)* |
| Digital literacy and comfort | *“Some people don't know how to use phones in the prison, so getting out might be weird for them to do this because they're not used to that.” (Participant 031, 31M)* |
| Privacy and security concerns | *“I know it's secure but I still feel like anyone can tap in, hackers or geniuses.” (Participant 023, 33F)*  *I'm not going to lie. I would just be worried. My information, that's a big worry of mine these days. Especially—let's be honest—people do pull shady shit all the time… You could be online, next thing you know, all my personal information's gone.” (Participant 025, 26M)* |
| **Drivers to engaging in digital health** | |
| Access to quality devices and the Internet | “*It's definitely helpful. [A study phone] definitely gives you a year to get back on your feet before you have to start paying for a cell phone.” (Participant 023, 33F)* |
| Ease of use | *“It's a lot easier to do it on your phone. So, you don't have to make CTS rides [i.e., Medicaid-sponsored rides], you don't have to go.” (013, 23M)*  *“When I got out, I started scheduling my appointments through the apps, so my MAT and all that other stuff I used to schedule through the app… if I need a refill, I can go on there and I can do it without having to bother anybody.” (Participant 034, 30F)* |
| Integrates into High Competing Demands, Low Resource Context of Reentry without Added Burden | *“[Digital healthcare has] always been good. Sometimes I feel like it's better [than in-person], especially if it's with a primary and just a routine thing or your therapist, because you don't have to worry about being late or waiting in the waiting room forever. It's just a lot more convenient… and the travel. I have my license, but I don't have a car. So as much as CTS, the insurance ride, has been great and everything, sometimes they're late and then I have to wait at my doctor's appointment. And my doctor understands, but a lot of the nurses and stuff will give you attitude about being late. So, it's just nice to avoid all of that.” (Participant 005, 36F)*  *“[Offering healthcare] more on the smartphone than making us come to you, it's going to be more convenient. When [name]… told me about [telehealth SUD treatment], I could breathe, dude. Literally, it is so hard to get back and forth to that shit, man, when you first get out. It is so overwhelming. It still is. It's only been three months.” (Participant 021, 36F)* |
